# Effect of acid suppression therapy on gastroesophageal reflux and cough in idiopathic pulmonary fibrosis: an intervention study

**DOI:** 10.1186/1745-9974-10-4

**Published:** 2014-04-30

**Authors:** Claire E Kilduff, Melanie J Counter, Gareth A Thomas, Nicholas K Harrison, Benjamin D Hope-Gill

**Affiliations:** 1 Department of Respiratory Medicine, Cardiff and Vale University Health Board, Cardiff, UK; 2 Department of Gastrointestinal Medicine, Cardiff and Vale University Health Board, Cardiff, UK; 3 College of Medicine, Swansea University, Swansea, UK

**Keywords:** Idiopathic pulmonary fibrosis, Gastroesophageal reflux, Cough, Anti-reflux therapy

## Abstract

**Background:**

Chronic cough affects more than 70 percent of patients with Idiopathic Pulmonary Fibrosis and causes significant morbidity. Gastroesophageal reflux is the cause of some cases of chronic cough; and also has a postulated role in the aetiology of Idiopathic Pulmonary Fibrosis. A high prevalence of acid; and more recently non-acid, reflux has been observed in Idiopathic Pulmonary Fibrosis cohorts. Therefore, gastroesophageal reflux may be implicated in the pathogenesis of cough in Idiopathic Pulmonary Fibrosis.

**Methods:**

Eighteen subjects with Idiopathic Pulmonary Fibrosis underwent 24-hour oesophageal impedance and cough count monitoring after the careful exclusion of causes of chronic cough other than gastroesophageal reflux. All 18 were then treated with high dose acid suppression therapies. Fourteen subjects underwent repeat 24-hour oesophageal impedance and cough count monitoring after eight weeks.

**Results:**

Total reflux and acid reflux frequencies were within the normal range in the majority of this cohort. The frequencies of non-acid and proximal reflux events were above the normal range. Following high dose acid suppression therapy there was a significant decrease in the number of acid reflux events (p = 0.02), but an increase in the number of non-acid reflux events (p = 0.01). There was no change in cough frequency (p = 0.70).

**Conclusions:**

This study confirms that non-acid reflux is prevalent; and that proximal oesophageal reflux occurs in the majority, of subjects with Idiopathic Pulmonary Fibrosis. It is the first study to investigate the effect of acid suppression therapy on gastroesophageal reflux and cough in patients with Idiopathic Pulmonary Fibrosis. The observation that cough frequency does not improve despite verifiable reductions in oesophageal acid exposure challenges the role of acid reflux in Idiopathic Pulmonary Fibrosis associated cough. The finding that non-acid reflux is increased following the use of acid suppression therapies cautions against the widespread use of acid suppression in patients with Idiopathic Pulmonary Fibrosis given the potential role for non-acid reflux in the pathogenesis of cough and Idiopathic Pulmonary Fibrosis itself.

**Study registration:**

The study was registered with the Cardiff and Vale University Local Health Board Research and Development Committee (09/CMC/4619) and the South East Wales Ethics Committee (09/WSE04/57).

## Background

Idiopathic pulmonary fibrosis (IPF) is a chronic, fibrotic interstitial lung disease of unknown origin associated with the histological and/or radiological pattern of usual interstitial pneumonia [1]. It typically presents with progressive breathlessness and more than 70% of patients also complain of a persistent, troublesome cough, which is resistant to conventional antitussive therapy [2]. Cough is an airway defence mechanism which is mediated via vagal sensory nerves that are mainly located within the central and proximal airways [3]. It is therefore interesting that cough should be such a prominent symptom in IPF which predominantly affects peripheral lung parenchyma. A recent study using 24-hour cough monitors has shown that patients with IPF have higher cough counts than both normal controls and asthmatics; and that the cough counts correlate well with subjective assessments of cough [4]. It has also been demonstrated that cough has a significant, detrimental effect on quality of life in patients with IPF [5].

The mechanisms underlying the pathogenesis of IPF remain unknown. However, one hypothesis proposes that chronic aspiration of gastric contents may cause repeated injury to alveolar epithelium resulting in the pathological abnormalities of usual interstitial pneumonia [6]. This hypothesis has recently received support from studies using 24-hour oesophageal pH monitoring and oesophageal manometry. The largest of these found that 87% of patients with IPF had abnormal acid gastroesophageal reflux (GOR) which was often clinically silent and occurred despite treatment with acid suppression therapy [7]. Furthermore, two small, retrospective, observational studies have reported clinical stabilisation of patients with IPF following strategies aimed at the management of GOR [8,9]. Consequently, international guidelines recommend that the majority of patients with IPF and asymptomatic acid reflux should receive medical treatment with acid suppression therapy, whilst acknowledging that the evidence to support such a recommendation is weak [10,11]. Despite the widespread use of acid suppression therapy in patients with IPF, the impact of such treatment on GOR and cough in this condition has not been investigated.

Gastroesophageal reflux has long been known to be an important cause of chronic cough [12]. Indeed a diagnosis of acid GOR-associated cough is often made following a successful, empirical trial of high-dose acid suppression therapy [13]. Thus, a particular attraction of the reflux hypothesis is that it could explain both the pathogenesis of IPF and the associated symptom of cough.

Despite the accumulating body of evidence linking IPF, cough and acid reflux, less is known about non-acid reflux in this context. A recent advance in the assessment of non-acid reflux is the technique of combined oesophageal pH and impedance monitoring. This provides a means of detecting and characterizing all reflux episodes regardless of pH [14]. Using this technique a recent study has shown a high prevalence of non-acid gastroesophageal reflux in patients with IPF compared with controls [15]. A study using pH-impedance monitoring in patients with systemic sclerosis, a condition complicated by oesophageal dysmotility and GOR, showed a positive relationship between the degree of acid and non-acid GOR and extent of pulmonary fibrosis estimated on CT scan [16].

Cough reflex sensitivity to capsaicin is known to be increased in IPF suggesting there is up-regulation of sensory C-fibres in the airways in IPF [17]. Cough reflex sensitivity to capsaicin is similarly increased in patients with both acid and non-acid reflux [18].

In this context, the objective of the present study was to investigate the impact of high-dose acid suppressant therapy on GOR and cough in this condition.

## Methods

### Subjects

We recruited 18 consecutive non-smoking subjects meeting the American Thoracic Society/European Respiratory Society criteria for the diagnosis of IPF [1]. Other causes of cough, except gastroesophageal reflux, were carefully excluded by clinical history, examination and investigation in accordance with national guidelines [13]. Subject characteristics are shown in Table 1. One-third of the cohort were taking immunosuppressive and immune-modulatory therapies at the time of the study, with some variation from triple therapy with prednisolone, N-acetylcysteine and azathioprine due to individual subject’s drug intolerances as detailed in Table 1.

**Table 1:** Subject characteristics

**Sex**	**5 F:13 M**
Age: Mean (95% CI)	65 (60–71)
FVC% predicted: Mean (95% CI)	83.1 (74.1-92.1)
TLCO% predicted: Mean (95% CI)	53.4 (44.8-62.1)
Proportion of subjects taking:	
Prednisolone	5/18
N-acetylcysteine	6/18
Azathioprine	5/18
Mycophenolate Mofetil	1/18

*Definition of abbreviations*: F = female; M = male; 95% CI = 95% percentile confidence interval.

### Combined 24 hour pH-impedance and 24 hour cough monitoring

All acid suppression and prokinetic medication was withheld for at least one week prior to study assessment. The 18 subjects underwent oesophageal manometry immediately followed by 24-hour combined pH-impedance monitoring (ZepHr, Sandhill Scientific, Highlands Ranch, CO). Impedance detected reflux events were defined according to pH as follows: acid reflux, pH <4; weakly acid reflux, pH 4 – 6.9; non-acid reflux, pH ≥ 7 [19]. Total reflux was calculated as the sum of acid, weakly acid and non-acid reflux events. The reference ranges used to define normal values of reflux events were those previously published for healthy populations [20]. Abnormal acid exposure was defined as pH <4 for ≥4.2% of the total study time [21]. The DeMeester score, a composite score based on the number and length of acid reflux episodes and the length of time that oesophageal pH <4 [22], was calculated automatically by the pH-impedance software. Simultaneous 24-hour cough monitoring was performed using a semi-automated cough detection system (Leicester Cough Monitor, Version 1.2, with kind permission of Dr Surinder Birring) [23]. Manometry and 24-hour pH-impedance results were analysed by two experienced operators blinded to the results of the 24-hour cough count. Operator input for the 24-hour cough counts was performed by a third operator blinded to the results of the manometry and pH-impedance as previously described [23].

All 18 subjects were then treated with open label high dose proton pump inhibitor therapy (omeprazole 40 mg twice daily or lansoprazole 30 mg twice daily) plus ranitidine 300 mg nocte for 8 weeks according to a protocol agreed a priori. Proton pump inhibition is endorsed by national and international guidelines as an empiric treatment for suspected GOR related cough [13,24]. Asymptomatic GOR is known to be prevalent amongst IPF cohorts as outlined above. Therefore, high dose proton pump inhibitor therapy was used to ensure adequate acid suppression and H2-receptor antagonism was included in the protocol to treat potential breakthrough nocturnal reflux [25]. Fourteen subjects agreed to undergo repeat combined 24-hour pH-impedance and 24-hour cough assessment following the 8 week treatment period, the other four subjects withdrew from the study prior to the second assessment. Three subjects also received additional prokinetic therapy (metoclopramide 10 mg three times daily or domperidone 10 mg three times daily).

### Statistical analysis

The Wilcoxon signed-rank test was used to compare paired non-parametric continuous data. The Kruskal-Wallis test was used to compare differences between non-parametric categorical variables. Proportions were compared using Fishers Exact Test. Spearman’s rank order correlation was used to assess correlation coefficients between non-parametrically distributed variables. All tests performed were two-sided tests at the 0.05 level of significance (SPSS version 18).

### Institutional approval

The study was approved by the South East Wales Ethics Committee (09/WSE04/57) and the Cardiff and Vale University Local Health Board Research and Development Committee (09/CMC/4619). Subjects provided written informed consent.

## Results

### Baseline 24-hour pH-impedance data

**Figure 1:**
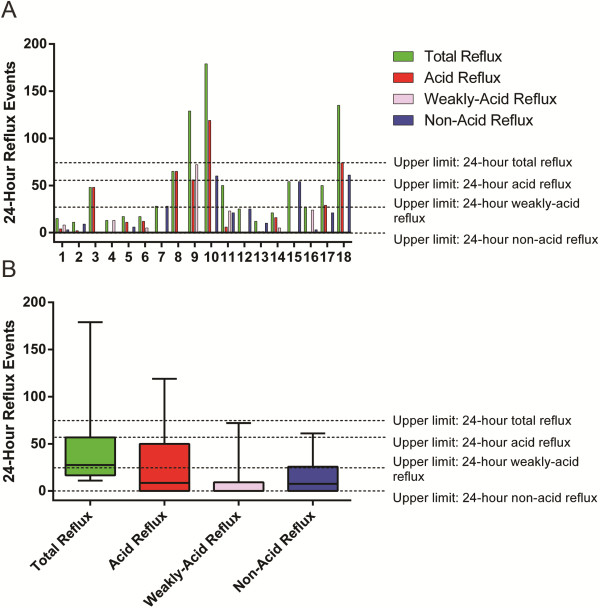
**Baseline 24-hour reflux events in 18 IPF subjects undergoing 24-hour pH-impedance monitoring.** A: Histogram and B: Box and whisker plot (median, quartiles and outlying values) (hatched lines = the upper limit of normal for each reflux subgroup [20]).

The spectrum of GOR events recorded for individual subjects is shown in Figure 1. The number of 24-hour total reflux events, 24-hour acid reflux events and 24-hour weakly acid reflux events were within normal limits for the majority of subjects (83% - 15/18, 78% - 14/18, 94% - 17/18 respectively). Acid exposure time was also within normal limits for just over half the subjects (56% - 10/18). Most subjects had a negative DeMeester score (61% - 11/18). Non-acid reflux was present in the majority of subjects (72% - 13/18). A high proportion of proximal reflux events (mean 74.7% - Table 2) and supine reflux events (median 11.7% - Table 3) were recorded in the study cohort as a whole.

**Table 2:** Proximal reflux events as percentage of total reflux events

**Subject number**	**Proximal reflux events as a % of total events**
1	86.7
2	72.7
3	81.3
4	53.8
5	100.0
6	35.3
7	78.6
8	43.8
9	58.1
10	100.0
11	44.0
12	84.0
13	91.7
14	52.4
15	100.0
16	63.0
17	100.0
18	100.0
	Mean 74.7

**Table 3:** Supine reflux events as percentage of total reflux events

**Subject number**	**Supine reflux events as a % of total events**
1	40.0
2	18.2
3	41.7
4	46.2
5	5.6
6	5.9
7	0.0
8	12.3
9	0.0
10	9.5
11	0.0
12	24.0
13	25.0
14	42.9
15	5.6
16	63.0
17	2.0
18	11.1
	Median 11.7

Lower oesophageal sphincter pressure was within normal limits for 61% (11/18) of subjects. There was a significant negative correlation between the number of total reflux events and lower oesophageal sphincter pressure (r = −0.68, p < 0.01). Manometry studies showed 17% (3/18) of subjects had evidence of ineffective oesophageal motility. No other indices of abnormal oesophageal motility were detected.

### Post treatment 24-hour pH-impedance data

**Figure 2:**
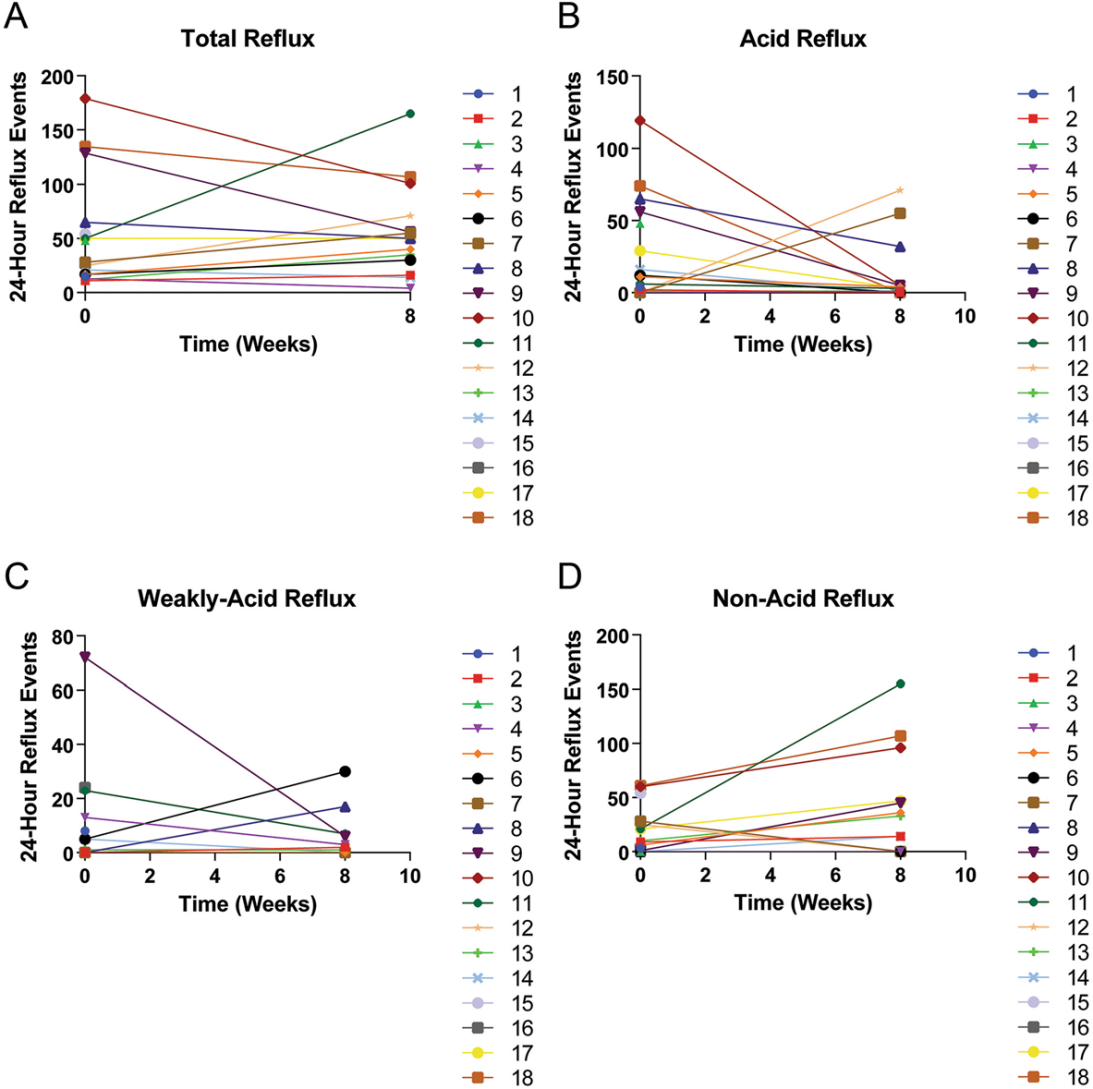
**24-hour reflux events before and after 8 weeks treatment with acid suppressant medication.** Line graphs of **A**: 24-hour total reflux **B**: 24-hour acid reflux **C**: 24-hour weakly acid reflux and **D**: 24-hour non-acid reflux.

Following eight weeks treatment with high dose acid suppression therapy the majority of subjects had a decrease in the number of acid reflux events (p = 0.02) and an increase in the number of non-acid reflux events (p = 0.01) (Table 4) (Figures 2A-D). In three subjects (subjects 4, 7 and 12) there was no reduction in acid reflux events and no associated increase in non-acid reflux events following acid suppression therapy. There was no significant difference between the number of 24-hour total reflux events before and after acid suppression therapy (p = 0.81; Figure 2).

**Table 4:** Change in number of reflux events following 8 weeks of acid suppressant therapy

	**Reflux event number**		
	**Increase**	**No change**	**Decrease**
Acid reflux	3	1	10
Non-acid reflux	9	3	2

### Post treatment 24-hour cough count data

**Figure 3:**
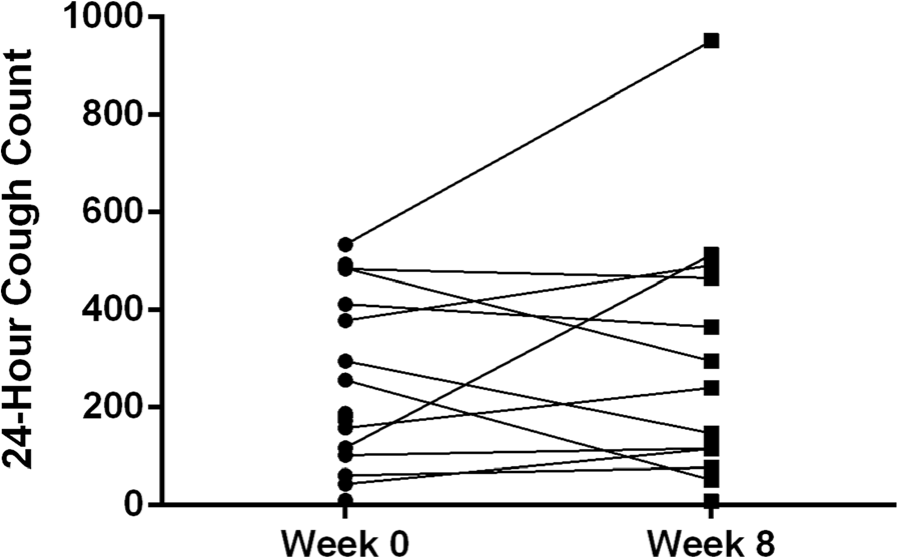
Line graph of 24-hour cough count before and after 8 weeks treatment with acid suppressant medication.

There was no significant change in 24 hour cough count (median; IQ Range) before (188; 338) and after (240; 382) acid suppression therapy (p = 0.70; Figure 3). There was no significant change in total reflux events (p = 0.18) or cough counts (p = 0.59) before and after therapy in the three subjects who also received prokinetic therapy.

## Discussion

This is the first study to investigate the effect of acid suppression therapy on gastroesophageal reflux and cough in patients with IPF. We have confirmed recent findings that non-acid reflux is prevalent and that proximal oesophageal reflux occurs in the majority of patients. This observation may be important because non-acid reflux (pH ≥7) is known to be rare in healthy individuals [20,26].

A potential mechanism for chronic cough in patients with IPF and GOR is enhancement of cough reflex sensitivity due to neuronal changes within the airways induced by gastroesophageal refluxate. It has been demonstrated that induced sputum from patients with IPF contains higher levels of the neurotrophins, nerve growth factor and brain-derived neurotrophic factor, compared with normal controls [17]. Furthermore, a study investigating pulmonary neurotrophin expression in patients with idiopathic interstitial pneumonia found evidence of enhanced expression of neurotrophins in the lungs of patients with IPF compared to other interstitial lung diseases, with fibroblastic foci in particular showing intense immunostaining for brain-derived neurotrophic factor and its receptor TrkB [27]. The cause of the enhanced neurotrophin expression in the lungs and airways of patients with IPF is unknown; however these observations raise the possibility that the neurotrophins may influence neuronal proliferation and differentiation in more proximal airways.

Gastric juice is a complex mixture of gastric acid, gastric enzymes such as pepsin; and bile acids and pancreatic enzymes from the duodenum. Hence, it may be of acid, neutral or alkaline pH. The pH of refluxate influences its biochemical activity and cellular toxicity. For example, animal studies have shown that within an acidic milieu pepsin potentiates the degree of acid-induced injury to oesophageal epithelial cells, but that at neutral pH pepsin is inactive and its oesophageal epithelial toxicity is ameliorated [28]. The toxicity of individual bile acids also varies with pH and conjugation. Animal studies have shown that conjugated bile acids cause injury to the oesophageal mucosa at acidic pH, whereas unconjugated bile acids are more injurious at a neutral pH [29]. In-vitro studies have shown that human airway epithelial cells exposed to unconjugated bile salts produce increased levels of the fibrogenic cytokine Transforming Growth Factor-β1 [30]. There is also preliminary data to suggest that airway epithelial cells exposed to pepsin can undergo epithelial-mesenchymal transformation, a process which is implicated in the pathogenesis of IPF [31]. In this context, our finding of frequent proximal reflux events in IPF subjects might be important because it demonstrates a potential mechanism by which alveolar epithelial cells could come into contact with the many components of gastric juice outlined above. A rodent model of chronic aspiration has shown that lung injury was caused by pH-neutral gastric constituents but not by hydrochloric acid [32]. Therefore, it is possible that incremental lung injury caused by chronic microaspiration is due to the non-acid components of gastric juice.

Supine reflux from the stomach into the oesophagus is rare in normal human subjects [20,26] probably because lower oesophageal sphincter relaxations are uncommon during sleep [33]. By contrast, reflux from the duodenum into the stomach is a normal, physiological, postprandial event and it also occurs nocturnally [29]. A previous study in patients with GORD found increased levels of bile acids in oesophageal aspirates compared with normal controls, particularly postprandially and in the supine position [34]. Hence, it is also of interest that reflux occurred in the supine position in the majority of our cohort.

As might be predicted, the present study objectively demonstrated a marked decrease in acid reflux following treatment with high-dose proton pump inhibitors (PPIs) and H2-receptor antagonists in the majority of patients with IPF. However, an unexpected finding was that this was consistently associated with an increase in non-acid reflux. Proton pump inhibitors are widely prescribed for acid suppression therapy in patients with IPF; therefore our findings that increased non-acid reflux may be a consequence of PPI therapy in such patients is an important observation.

The effect of PPIs on duodeno-gastroesophageal reflux is complex. Whilst they reduce gastric volume, which should reduce reflux into the oesophagus, they also delay gastric emptying which might enhance reflux [35]. It has been shown that PPIs have no effect on levels of pepsin or bile acids in bronchoalveolar lavage from patients after lung transplantation, despite reducing oesophageal acid exposure [36]. We were careful to exclude other known causes of cough in this patient cohort. Therefore, our observation that cough can occur despite verifying low levels of oesophageal acidity; and that cough counts do not diminish following high dose acid suppression therapy, suggests that acid-GOR per se is not the sole cause of cough in IPF. This finding is in keeping with negative randomized, controlled trials of acid suppression therapy in patients with GOR and chronic cough [37,38].

The exclusion of confounding causes of cough and the invasive nature of the investigations performed has limited the size of the study cohort and also resulted in a high withdrawal rate for the second pH-impedance assessment. A combination of the small sample size and day to day variations in reflux frequency [39] may explain the unexpectedly low rates of acid reflux observed in our population compared with previous larger studies [7,40]. In addition, a previous study has shown that objective cough frequency and subjective cough severity are both reduced by the presence of an oesophageal catheter [41]. Such an inhibitory effect may have influenced the detection of a difference in cough count before and after acid suppression therapy. However, given that an oesophageal catheter was in situ both at baseline and eight weeks in the present study and the absence of a trend towards reduced cough after acid suppression therapy a type two error of this nature seems unlikely.

Despite these limitations, the careful subject selection process means that relevant observations about the relationship between cough, gastroesophageal reflux and acid suppression therapy in this condition can be made. The invasive nature of the investigations also made attempts to recruit a suitable control group unrewarding. However, the normal ranges for impedance detected reflux parameters have been well defined in both North American [20] and European populations [26]. Three subjects were also treated with prokinetic therapy and whilst no clear evidence of additional effect was observed, the group is too small to draw conclusions from.

## Conclusions

In conclusion, proximal, non-acid reflux is prevalent in patients with IPF and may be implicated in both the pathogenesis of pulmonary fibrosis and sensitisation of the cough reflex in this condition. The lack of improvement in cough symptoms despite effective acid suppression therapy indicates that acid GOR reflux alone is not responsible for cough in the majority of patients with IPF. The finding that acid suppression consistently increases the frequency of non-acid reflux cautions against the widespread empirical use of acid suppression in IPF and GOR in the absence of objective pH or impedance testing. Our findings suggest that strategies for treating GOR in patients with IPF should target both acid and non-acid reflux, perhaps by including prokinetic therapies, rather than acid suppression therapy alone.

## Abbreviations

GOR: Gastroesophageal reflux; IPF: Idiopathic pulmonary fibrosis; PPI: Proton pump inhibitor.

## Competing interests

The authors declare that they have no competing interests.

## Authors’ contributions

CEK: conception and design of study; acquisition, analysis and interpretation of data; drafting, critical revision and final approval of manuscript. MJC: acquisition, analysis and interpretation of data; final approval of manuscript. GAT: acquisition, analysis and interpretation of data; drafting, critical revision and final approval of manuscript. NKH: conception and design of study; drafting, critical revision and final approval of manuscript. BHG: conception and design of study; acquisition, analysis and interpretation of data; drafting, critical revision and final approval of manuscript.

## Authors’ information

CEK is a clinical research fellow and specialty trainee in respiratory medicine.

MJC is a gastro-intestinal physiologist. GAT is a consultant in general and gastrointestinal medicine with an interest in gastroesophageal reflux. NKH is a consultant in general and respiratory medicine with an interest in interstitial lung disease. BHG is consultant in general and respiratory medicine with an interest in interstitial lung disease.
